# 5′′-Benzyl­idene-1′′-methyl-1′-phenyl-1′,2′,3′,5′,6′,7′,8′,8a’-octa­hydro­dispiro[acenaphthyl­ene-1,3′-indolizine-2′,3′′-piperidine]-2,4′′(1*H*)-dione

**DOI:** 10.1107/S1600536812039591

**Published:** 2012-09-22

**Authors:** J. Suresh, R. A. Nagalakshmi, R. Ranjith Kumar, S. Sivakumar, P. L. Nilantha Lakshman

**Affiliations:** a Department of Physics, The Madura College, Madurai 625 011, India; b Department of Organic Chemistry, School of Chemistry, Madurai Kamaraj University, Madurai 625 021, India; c Department of Food Science and Technology, University of Ruhuna, Mapalana, Kamburupitiya 81100, Sri Lanka

## Abstract

In the title compound, C_37_H_34_N_2_O_2_, the pyridinone ring adopts a half-chair conformation. In the octa­hydro­indolizine fused-ring system, the piperidine ring is in a chair conformation and the pyrrole ring is twisted about the N—C(piperidine) bond. The mol­ecular structure features a weak intra­molecular C—H⋯O inter­action.

## Related literature

For the importance of spiro compounds, see: Gubin *et al.* (1992 ▶); Liu *et al.* (2007 ▶); Molyneux & James (1982 ▶); Nash *et al.* (1988 ▶); Pearson & Guo (2001 ▶); Smith *et al.* (2007 ▶). For related acenaphthyl­ene structures, see: Sundar *et al.* (2002 ▶). For additional conformational analysis, see: Cremer & Pople (1975 ▶).
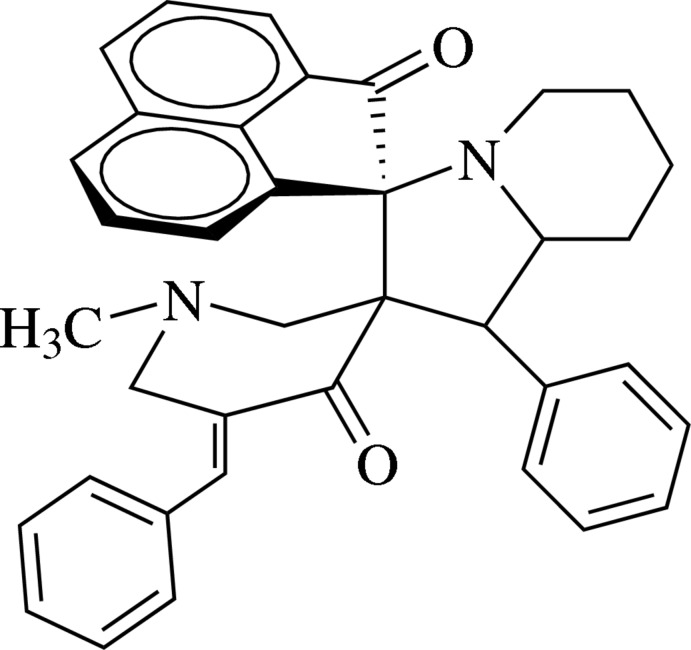


## Experimental

### 

#### Crystal data

C_37_H_34_N_2_O_2_
*M* *_r_* = 538.66
Monoclinic, 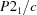

*a* = 8.4913 (3) Å
*b* = 16.6782 (6) Å
*c* = 20.5435 (7) Å
β = 100.028 (2)°
*V* = 2864.92 (17) Å^3^
*Z* = 4
Mo *K*α radiation
μ = 0.08 mm^−1^
*T* = 293 K
0.21 × 0.19 × 0.18 mm

#### Data collection

Bruker Kappa APEXII diffractometer
Absorption correction: multi-scan (*SADABS*; Sheldrick, 1996 ▶) *T*_min_ = 0.967, *T*_max_ = 0.974
43034 measured reflections
9332 independent reflections
5857 reflections with *I* > 2σ(*I*)
*R*_int_ = 0.035

#### Refinement

*R*[*F* ^2^ > 2σ(*F* ^2^)] = 0.053
*wR*(*F* ^2^) = 0.157
*S* = 1.03
9332 reflections
371 parameters
H-atom parameters constrained
Δρ_max_ = 0.29 e Å^−3^
Δρ_min_ = −0.23 e Å^−3^

### 

Data collection: *APEX2* (Bruker, 2004 ▶); cell refinement: *SAINT* (Bruker, 2004 ▶); data reduction: *SAINT*; program(s) used to solve structure: *SHELXS97* (Sheldrick, 2008 ▶); program(s) used to refine structure: *SHELXL97* (Sheldrick, 2008 ▶); molecular graphics: *PLATON* (Spek, 2009 ▶); software used to prepare material for publication: *SHELXL97*.

## Supplementary Material

Crystal structure: contains datablock(s) global, I. DOI: 10.1107/S1600536812039591/tk5152sup1.cif

Structure factors: contains datablock(s) I. DOI: 10.1107/S1600536812039591/tk5152Isup2.hkl

Additional supplementary materials:  crystallographic information; 3D view; checkCIF report

## Figures and Tables

**Table 1:** Hydrogen-bond geometry (Å, °)

*D*—H⋯*A*	*D*—H	H⋯*A*	*D*⋯*A*	*D*—H⋯*A*
C2—H2*B*⋯O2	0.97	2.35	2.9329 (18)	118

**Fig. 1.**
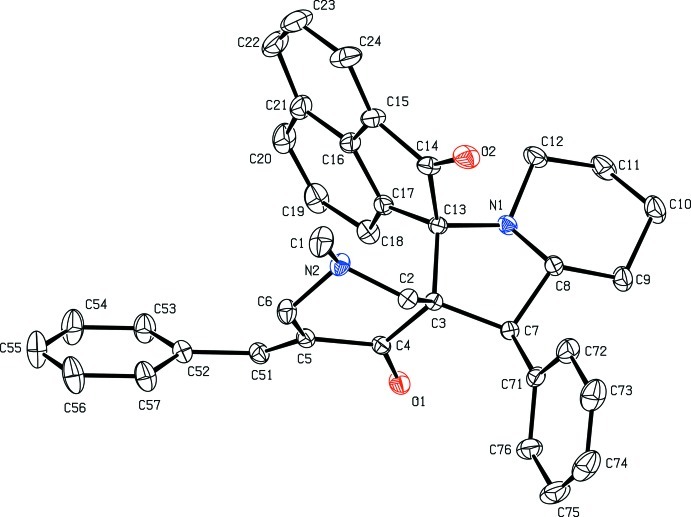
The molecular structure of (I), showing 20% probability displacement ellipsoids and the atom-numbering scheme. H-atoms are omitted for clarity.

## supplementary crystallographic information

### Comment

Spiro indolizine derivatives have been found to possess a variety of biological activities such as antibacterial, antiinflammatory, antiviral, (Nash *et al.*, 1988; Molyneux & James, 1982), anticancer (Liu *et al.*, 2007; Smith *et al.*, 2007) and antitumor (Pearson & Guo, 2001). They are also important synthetic targets in view of developing new pharmaceuticals for the treatment of cardiovascular diseases (Gubin *et al.*, 1992). In view of the high medicinal value of these compounds in conjunction with our research interests, prompted us to synthesize and report the X-ray studies of the title compound. Related acenaphthylene structures are known (Sundar *et al.*, 2002).

In the title compound (Fig. 1), the pyridinone ring adopts twisted chair conformation with atoms N2 and C3 deviating by -0.6237 (1) and -0.4716 (1) Å, respectively, from the least-squares plane defined by other atoms (C2/C4/C5/C6). Within the octahydroindolizine, the six membered piperidine ring adopts chair conformation as evident from the puckering parameters (Cremer & Pople, 1975): Q = 0.551 (2) Å, θ = 145.92 (2)° and Φ = 223.5 (3)°. The dihedral angle between the two benzene rings and the acenapthalene rings are 70.74 (1) and 34.81 (1) Å. The molecular structure also features a weak intramolecular C—H···O interaction (Table 1).

### Experimental

A mixture of 1-methyl-3,5-bis[(*E*)-arylmethylidene]tetrahydro-4(1*H*)-pyridinone (1 mmol), acenaphthenequinone (1 mmol) and piperidine-2-carboxylic acid (1 mmol) was dissolved in isopropyl alcohol (15 ml), and heated to reflux for 60 min. After completion of the reaction, as evident from TLC, the mixture was poured into water (50 ml), the precipitated solid was filtered and washed with water (100 ml) to obtain pure yellow solid. Melting point: 479 K, Yield: 89%

### Refinement

H atoms were placed at calculated positions and allowed to ride on their carrier atoms with C—H = 0.93–0.98 Å and *U*_iso_ = 1.2–1.5*U*_eq_(C).

### Crystal data

**Table:**

C_37_H_34_N_2_O_2_	*F*(000) = 1144
*M**_r_* = 538.66	*D*_x_ = 1.249 Mg m^−^^3^
Monoclinic, *P*2_1_/*c*	Mo *K*α radiation, λ = 0.71073 Å
Hall symbol: -P 2ybc	Cell parameters from 2000 reflections
*a* = 8.4913 (3) Å	θ = 2–31°
*b* = 16.6782 (6) Å	µ = 0.08 mm^−^^1^
*c* = 20.5435 (7) Å	*T* = 293 K
β = 100.028 (2)°	Block, yellow
*V* = 2864.92 (17) Å^3^	0.21 × 0.19 × 0.18 mm
*Z* = 4	

### Data collection

**Table:**

Bruker Kappa APEXII diffractometer	9332 independent reflections
Radiation source: fine-focus sealed tube	5857 reflections with *I* > 2σ(*I*)
Graphite monochromator	*R*_int_ = 0.035
Detector resolution: 0 pixels mm^-1^	θ_max_ = 31.3°, θ_min_ = 1.6°
ω and φ scans	*h* = −12→12
Absorption correction: multi-scan (*SADABS*; Sheldrick, 1996)	*k* = −22→24
*T*_min_ = 0.967, *T*_max_ = 0.974	*l* = −25→30
43034 measured reflections	

### Refinement

**Table:**

Refinement on *F*^2^	Secondary atom site location: difference Fourier map
Least-squares matrix: full	Hydrogen site location: inferred from neighbouring sites
*R*[*F*^2^ > 2σ(*F*^2^)] = 0.053	H-atom parameters constrained
*wR*(*F*^2^) = 0.157	*w* = 1/[σ^2^(*F*_o_^2^) + (0.0678*P*)^2^ + 0.5825*P*] where *P* = (*F*_o_^2^ + 2*F*_c_^2^)/3
*S* = 1.03	(Δ/σ)_max_ < 0.001
9332 reflections	Δρ_max_ = 0.29 e Å^−^^3^
371 parameters	Δρ_min_ = −0.23 e Å^−^^3^
0 restraints	Extinction correction: *SHELXL97* (Sheldrick, 2008), Fc^*^=kFc[1+0.001xFc^2^λ^3^/sin(2θ)]^-1/4^
Primary atom site location: structure-invariant direct methods	Extinction coefficient: 0.0030 (7)

### Special details

**Table:**

Geometry. All e.s.d.'s (except the e.s.d. in the dihedral angle between two l.s. planes) are estimated using the full covariance matrix. The cell e.s.d.'s are taken into account individually in the estimation of e.s.d.'s in distances, angles and torsion angles; correlations between e.s.d.'s in cell parameters are only used when they are defined by crystal symmetry. An approximate (isotropic) treatment of cell e.s.d.'s is used for estimating e.s.d.'s involving l.s. planes.
Refinement. Refinement of *F*^2^ against ALL reflections. The weighted *R*-factor *wR* and goodness of fit *S* are based on *F*^2^, conventional *R*-factors *R* are based on *F*, with *F* set to zero for negative *F*^2^. The threshold expression of *F*^2^ > σ(*F*^2^) is used only for calculating *R*-factors(gt) *etc*. and is not relevant to the choice of reflections for refinement. *R*-factors based on *F*^2^ are statistically about twice as large as those based on *F*, and *R*- factors based on ALL data will be even larger.

### Fractional atomic coordinates and isotropic or equivalent isotropic displacement parameters (Å^2^)

**Table:**

	*x*	*y*	*z*	*U*_iso_*/*U*_eq_	
O1	0.02236 (11)	0.45746 (6)	0.21716 (5)	0.0463 (3)	
N1	0.18434 (14)	0.27311 (6)	0.30171 (5)	0.0354 (2)	
C13	0.29204 (15)	0.32105 (7)	0.26884 (6)	0.0316 (2)	
C7	0.14839 (15)	0.39871 (7)	0.34350 (6)	0.0311 (2)	
H7	0.0373	0.4034	0.3208	0.037*	
N2	0.50761 (13)	0.45681 (7)	0.26318 (6)	0.0386 (3)	
C4	0.16715 (15)	0.45441 (7)	0.22914 (6)	0.0320 (3)	
C71	0.16991 (16)	0.45865 (8)	0.39942 (7)	0.0370 (3)	
O2	0.54309 (13)	0.29695 (7)	0.34803 (6)	0.0555 (3)	
C2	0.40959 (15)	0.45688 (8)	0.31454 (6)	0.0342 (3)	
H2A	0.3840	0.5115	0.3250	0.041*	
H2B	0.4676	0.4321	0.3543	0.041*	
C17	0.26458 (17)	0.29985 (8)	0.19575 (6)	0.0364 (3)	
C51	0.19570 (16)	0.50244 (8)	0.12120 (7)	0.0383 (3)	
H51	0.0901	0.4852	0.1108	0.046*	
C3	0.25581 (14)	0.41018 (7)	0.28993 (6)	0.0291 (2)	
C52	0.26203 (17)	0.53783 (8)	0.06646 (7)	0.0406 (3)	
C18	0.1305 (2)	0.30038 (9)	0.14857 (7)	0.0452 (3)	
H18	0.0337	0.3186	0.1582	0.054*	
C14	0.47326 (17)	0.29755 (8)	0.29136 (7)	0.0415 (3)	
C8	0.17403 (16)	0.31133 (7)	0.36444 (6)	0.0341 (3)	
H8	0.2759	0.3056	0.3949	0.041*	
C5	0.26617 (15)	0.49163 (8)	0.18385 (7)	0.0342 (3)	
C9	0.0405 (2)	0.27382 (9)	0.39425 (8)	0.0470 (3)	
H9A	−0.0615	0.2847	0.3662	0.056*	
H9B	0.0385	0.2972	0.4373	0.056*	
C6	0.43879 (16)	0.51107 (10)	0.21008 (7)	0.0432 (3)	
H6A	0.4997	0.5074	0.1744	0.052*	
H6B	0.4466	0.5657	0.2264	0.052*	
C15	0.53287 (19)	0.26629 (9)	0.23289 (8)	0.0485 (4)	
C21	0.4175 (2)	0.23699 (10)	0.11699 (9)	0.0558 (4)	
C16	0.40641 (19)	0.26788 (8)	0.17969 (7)	0.0428 (3)	
C12	0.2153 (2)	0.18695 (8)	0.30668 (8)	0.0488 (4)	
H12A	0.3173	0.1771	0.3350	0.059*	
H12B	0.2199	0.1652	0.2633	0.059*	
C72	0.2823 (2)	0.44916 (10)	0.45633 (7)	0.0521 (4)	
H72	0.3500	0.4049	0.4603	0.063*	
C11	0.0826 (2)	0.14647 (9)	0.33507 (9)	0.0571 (4)	
H11A	0.1059	0.0897	0.3409	0.068*	
H11B	−0.0174	0.1520	0.3044	0.068*	
C10	0.0659 (2)	0.18347 (10)	0.40116 (9)	0.0592 (4)	
H10A	0.1616	0.1727	0.4333	0.071*	
H10B	−0.0242	0.1593	0.4171	0.071*	
C1	0.67269 (18)	0.47886 (13)	0.28887 (9)	0.0613 (5)	
H1A	0.7335	0.4781	0.2536	0.092*	
H1B	0.7177	0.4413	0.3224	0.092*	
H1C	0.6757	0.5317	0.3075	0.092*	
C76	0.0721 (2)	0.52561 (9)	0.39544 (9)	0.0537 (4)	
H76	−0.0048	0.5340	0.3579	0.064*	
C20	0.2790 (3)	0.24183 (11)	0.06903 (9)	0.0658 (5)	
H20	0.2808	0.2239	0.0263	0.079*	
C53	0.2212 (2)	0.50518 (11)	0.00374 (8)	0.0577 (4)	
H53	0.1538	0.4609	−0.0027	0.069*	
C57	0.3575 (2)	0.60591 (10)	0.07381 (8)	0.0565 (4)	
H57	0.3839	0.6302	0.1150	0.068*	
C75	0.0883 (3)	0.58062 (11)	0.44760 (13)	0.0743 (6)	
H75	0.0218	0.6253	0.4443	0.089*	
C19	0.1418 (3)	0.27253 (11)	0.08442 (8)	0.0600 (5)	
H19	0.0517	0.2754	0.0515	0.072*	
C74	0.1995 (3)	0.56989 (13)	0.50282 (11)	0.0770 (6)	
H74	0.2095	0.6070	0.5371	0.092*	
C23	0.6913 (3)	0.20440 (14)	0.16253 (14)	0.0856 (7)	
H23	0.7891	0.1832	0.1564	0.103*	
C22	0.5681 (3)	0.20404 (12)	0.11094 (12)	0.0762 (6)	
H22	0.5826	0.1818	0.0709	0.091*	
C54	0.2788 (3)	0.53737 (14)	−0.04922 (9)	0.0760 (6)	
H54	0.2510	0.5143	−0.0908	0.091*	
C73	0.2965 (3)	0.50437 (13)	0.50781 (9)	0.0701 (5)	
H73	0.3724	0.4965	0.5457	0.084*	
C56	0.4135 (3)	0.63776 (13)	0.02018 (10)	0.0766 (6)	
H56	0.4772	0.6834	0.0256	0.092*	
C24	0.6777 (2)	0.23534 (12)	0.22476 (12)	0.0723 (6)	
H24	0.7643	0.2349	0.2594	0.087*	
C55	0.3763 (3)	0.60290 (15)	−0.04099 (10)	0.0802 (6)	
H55	0.4172	0.6238	−0.0766	0.096*	

### Atomic displacement parameters (Å^2^)

**Table:**

	*U*^11^	*U*^22^	*U*^33^	*U*^12^	*U*^13^	*U*^23^
O1	0.0309 (5)	0.0548 (6)	0.0527 (6)	0.0048 (4)	0.0061 (4)	0.0185 (5)
N1	0.0465 (6)	0.0259 (5)	0.0339 (6)	−0.0008 (4)	0.0076 (5)	0.0001 (4)
C13	0.0351 (6)	0.0294 (6)	0.0300 (6)	0.0038 (5)	0.0046 (5)	0.0002 (5)
C7	0.0313 (6)	0.0301 (6)	0.0326 (6)	−0.0001 (5)	0.0071 (5)	0.0008 (5)
N2	0.0276 (5)	0.0515 (7)	0.0370 (6)	−0.0019 (5)	0.0061 (4)	0.0012 (5)
C4	0.0322 (6)	0.0282 (6)	0.0354 (6)	0.0007 (5)	0.0053 (5)	0.0028 (5)
C71	0.0393 (7)	0.0339 (6)	0.0418 (7)	−0.0039 (5)	0.0187 (6)	−0.0039 (5)
O2	0.0497 (6)	0.0619 (7)	0.0496 (6)	0.0142 (5)	−0.0059 (5)	0.0064 (5)
C2	0.0321 (6)	0.0369 (6)	0.0334 (6)	−0.0024 (5)	0.0053 (5)	−0.0029 (5)
C17	0.0480 (8)	0.0294 (6)	0.0323 (6)	0.0001 (5)	0.0084 (6)	−0.0011 (5)
C51	0.0381 (7)	0.0348 (6)	0.0419 (7)	−0.0028 (5)	0.0067 (6)	0.0050 (5)
C3	0.0294 (6)	0.0282 (6)	0.0296 (6)	0.0012 (4)	0.0052 (4)	0.0013 (4)
C52	0.0433 (7)	0.0400 (7)	0.0381 (7)	−0.0026 (6)	0.0058 (6)	0.0064 (6)
C18	0.0546 (9)	0.0409 (7)	0.0377 (7)	−0.0058 (6)	0.0011 (6)	−0.0028 (6)
C14	0.0409 (7)	0.0368 (7)	0.0452 (8)	0.0097 (6)	0.0029 (6)	0.0011 (6)
C8	0.0413 (7)	0.0306 (6)	0.0303 (6)	−0.0015 (5)	0.0061 (5)	0.0011 (5)
C5	0.0342 (6)	0.0307 (6)	0.0386 (7)	0.0000 (5)	0.0084 (5)	0.0047 (5)
C9	0.0574 (9)	0.0417 (8)	0.0443 (8)	−0.0093 (7)	0.0154 (7)	0.0031 (6)
C6	0.0371 (7)	0.0523 (8)	0.0405 (7)	−0.0079 (6)	0.0081 (6)	0.0061 (6)
C15	0.0483 (8)	0.0409 (8)	0.0582 (9)	0.0112 (6)	0.0149 (7)	−0.0029 (7)
C21	0.0809 (12)	0.0408 (8)	0.0534 (10)	−0.0052 (8)	0.0325 (9)	−0.0091 (7)
C16	0.0557 (9)	0.0323 (7)	0.0441 (8)	0.0006 (6)	0.0193 (7)	−0.0041 (6)
C12	0.0672 (10)	0.0288 (7)	0.0497 (8)	0.0032 (6)	0.0084 (7)	0.0017 (6)
C72	0.0644 (10)	0.0537 (9)	0.0395 (8)	−0.0023 (7)	0.0124 (7)	−0.0090 (7)
C11	0.0745 (11)	0.0309 (7)	0.0644 (11)	−0.0082 (7)	0.0079 (9)	0.0046 (7)
C10	0.0774 (12)	0.0429 (8)	0.0602 (10)	−0.0139 (8)	0.0199 (9)	0.0114 (7)
C1	0.0307 (7)	0.0949 (14)	0.0573 (10)	−0.0090 (8)	0.0047 (7)	0.0078 (9)
C76	0.0509 (9)	0.0394 (8)	0.0754 (11)	0.0024 (6)	0.0239 (8)	−0.0058 (7)
C20	0.1035 (16)	0.0572 (10)	0.0409 (9)	−0.0175 (10)	0.0240 (10)	−0.0153 (7)
C53	0.0724 (11)	0.0551 (10)	0.0472 (9)	−0.0198 (8)	0.0150 (8)	−0.0028 (7)
C57	0.0699 (11)	0.0535 (9)	0.0444 (8)	−0.0209 (8)	0.0047 (8)	0.0056 (7)
C75	0.0807 (14)	0.0449 (9)	0.1095 (18)	0.0002 (9)	0.0502 (13)	−0.0219 (10)
C19	0.0859 (13)	0.0544 (10)	0.0361 (8)	−0.0158 (9)	0.0005 (8)	−0.0064 (7)
C74	0.0941 (15)	0.0696 (13)	0.0778 (14)	−0.0238 (11)	0.0442 (13)	−0.0385 (11)
C23	0.0820 (15)	0.0721 (14)	0.115 (2)	0.0208 (11)	0.0528 (15)	−0.0141 (13)
C22	0.1001 (17)	0.0591 (11)	0.0839 (15)	0.0003 (11)	0.0566 (14)	−0.0189 (10)
C54	0.1030 (16)	0.0851 (15)	0.0436 (10)	−0.0282 (12)	0.0228 (10)	−0.0055 (9)
C73	0.0879 (14)	0.0775 (13)	0.0477 (10)	−0.0191 (11)	0.0194 (9)	−0.0225 (9)
C56	0.0903 (15)	0.0769 (13)	0.0618 (12)	−0.0402 (11)	0.0107 (10)	0.0169 (10)
C24	0.0583 (11)	0.0675 (12)	0.0946 (15)	0.0221 (9)	0.0227 (10)	−0.0086 (11)
C55	0.0961 (16)	0.0948 (16)	0.0544 (11)	−0.0282 (13)	0.0259 (11)	0.0160 (11)

### Geometric parameters (Å, º)

**Table:**

O1—C4	1.2122 (15)	C15—C16	1.393 (2)
N1—C8	1.4539 (16)	C21—C20	1.399 (3)
N1—C12	1.4612 (18)	C21—C16	1.406 (2)
N1—C13	1.4652 (17)	C21—C22	1.418 (3)
C13—C17	1.5207 (17)	C12—C11	1.514 (2)
C13—C14	1.5772 (18)	C12—H12A	0.9700
C13—C3	1.5933 (17)	C12—H12B	0.9700
C7—C71	1.5097 (18)	C72—C73	1.391 (2)
C7—C8	1.5243 (17)	C72—H72	0.9300
C7—C3	1.5588 (16)	C11—C10	1.520 (3)
C7—H7	0.9800	C11—H11A	0.9700
N2—C2	1.4533 (16)	C11—H11B	0.9700
N2—C1	1.4564 (18)	C10—H10A	0.9700
N2—C6	1.4589 (18)	C10—H10B	0.9700
C4—C5	1.4934 (17)	C1—H1A	0.9600
C4—C3	1.5306 (17)	C1—H1B	0.9600
C71—C72	1.384 (2)	C1—H1C	0.9600
C71—C76	1.386 (2)	C76—C75	1.399 (3)
O2—C14	1.2112 (17)	C76—H76	0.9300
C2—C3	1.5289 (17)	C20—C19	1.359 (3)
C2—H2A	0.9700	C20—H20	0.9300
C2—H2B	0.9700	C53—C54	1.377 (2)
C17—C18	1.360 (2)	C53—H53	0.9300
C17—C16	1.408 (2)	C57—C56	1.380 (2)
C51—C5	1.3337 (19)	C57—H57	0.9300
C51—C52	1.4666 (19)	C75—C74	1.355 (3)
C51—H51	0.9300	C75—H75	0.9300
C52—C53	1.386 (2)	C19—H19	0.9300
C52—C57	1.388 (2)	C74—C73	1.361 (3)
C18—C19	1.416 (2)	C74—H74	0.9300
C18—H18	0.9300	C23—C22	1.353 (3)
C14—C15	1.477 (2)	C23—C24	1.402 (3)
C8—C9	1.5144 (19)	C23—H23	0.9300
C8—H8	0.9800	C22—H22	0.9300
C5—C6	1.5064 (19)	C54—C55	1.364 (3)
C9—C10	1.525 (2)	C54—H54	0.9300
C9—H9A	0.9700	C73—H73	0.9300
C9—H9B	0.9700	C56—C55	1.371 (3)
C6—H6A	0.9700	C56—H56	0.9300
C6—H6B	0.9700	C24—H24	0.9300
C15—C24	1.371 (2)	C55—H55	0.9300
			
C8—N1—C12	114.03 (11)	C20—C21—C16	116.24 (16)
C8—N1—C13	107.94 (10)	C20—C21—C22	128.43 (17)
C12—N1—C13	116.74 (11)	C16—C21—C22	115.33 (19)
N1—C13—C17	109.12 (10)	C15—C16—C21	123.14 (15)
N1—C13—C14	112.62 (10)	C15—C16—C17	113.43 (13)
C17—C13—C14	101.88 (10)	C21—C16—C17	123.37 (15)
N1—C13—C3	102.61 (9)	N1—C12—C11	109.19 (13)
C17—C13—C3	118.80 (10)	N1—C12—H12A	109.8
C14—C13—C3	112.14 (10)	C11—C12—H12A	109.8
C71—C7—C8	115.21 (11)	N1—C12—H12B	109.8
C71—C7—C3	116.61 (10)	C11—C12—H12B	109.8
C8—C7—C3	104.13 (10)	H12A—C12—H12B	108.3
C71—C7—H7	106.7	C71—C72—C73	121.46 (17)
C8—C7—H7	106.7	C71—C72—H72	119.3
C3—C7—H7	106.7	C73—C72—H72	119.3
C2—N2—C1	111.92 (12)	C12—C11—C10	110.76 (13)
C2—N2—C6	109.69 (11)	C12—C11—H11A	109.5
C1—N2—C6	110.45 (12)	C10—C11—H11A	109.5
O1—C4—C5	121.15 (11)	C12—C11—H11B	109.5
O1—C4—C3	121.55 (11)	C10—C11—H11B	109.5
C5—C4—C3	117.28 (10)	H11A—C11—H11B	108.1
C72—C71—C76	117.41 (14)	C11—C10—C9	110.58 (13)
C72—C71—C7	122.78 (12)	C11—C10—H10A	109.5
C76—C71—C7	119.79 (14)	C9—C10—H10A	109.5
N2—C2—C3	108.88 (10)	C11—C10—H10B	109.5
N2—C2—H2A	109.9	C9—C10—H10B	109.5
C3—C2—H2A	109.9	H10A—C10—H10B	108.1
N2—C2—H2B	109.9	N2—C1—H1A	109.5
C3—C2—H2B	109.9	N2—C1—H1B	109.5
H2A—C2—H2B	108.3	H1A—C1—H1B	109.5
C18—C17—C16	118.64 (13)	N2—C1—H1C	109.5
C18—C17—C13	131.87 (13)	H1A—C1—H1C	109.5
C16—C17—C13	109.21 (12)	H1B—C1—H1C	109.5
C5—C51—C52	128.80 (13)	C71—C76—C75	120.34 (18)
C5—C51—H51	115.6	C71—C76—H76	119.8
C52—C51—H51	115.6	C75—C76—H76	119.8
C2—C3—C4	107.15 (10)	C19—C20—C21	120.48 (15)
C2—C3—C7	113.58 (10)	C19—C20—H20	119.8
C4—C3—C7	111.72 (10)	C21—C20—H20	119.8
C2—C3—C13	111.78 (10)	C54—C53—C52	121.02 (16)
C4—C3—C13	108.58 (10)	C54—C53—H53	119.5
C7—C3—C13	104.00 (9)	C52—C53—H53	119.5
C53—C52—C57	117.99 (14)	C56—C57—C52	120.26 (16)
C53—C52—C51	119.16 (13)	C56—C57—H57	119.9
C57—C52—C51	122.75 (14)	C52—C57—H57	119.9
C17—C18—C19	118.50 (16)	C74—C75—C76	121.05 (18)
C17—C18—H18	120.8	C74—C75—H75	119.5
C19—C18—H18	120.8	C76—C75—H75	119.5
O2—C14—C15	126.79 (13)	C20—C19—C18	122.66 (17)
O2—C14—C13	124.98 (13)	C20—C19—H19	118.7
C15—C14—C13	107.71 (12)	C18—C19—H19	118.7
N1—C8—C9	110.01 (11)	C75—C74—C73	119.54 (17)
N1—C8—C7	101.35 (10)	C75—C74—H74	120.2
C9—C8—C7	115.39 (11)	C73—C74—H74	120.2
N1—C8—H8	109.9	C22—C23—C24	122.73 (19)
C9—C8—H8	109.9	C22—C23—H23	118.6
C7—C8—H8	109.9	C24—C23—H23	118.6
C51—C5—C4	116.79 (12)	C23—C22—C21	121.06 (18)
C51—C5—C6	124.14 (12)	C23—C22—H22	119.5
C4—C5—C6	119.01 (11)	C21—C22—H22	119.5
C8—C9—C10	110.01 (13)	C55—C54—C53	120.40 (18)
C8—C9—H9A	109.7	C55—C54—H54	119.8
C10—C9—H9A	109.7	C53—C54—H54	119.8
C8—C9—H9B	109.7	C74—C73—C72	120.2 (2)
C10—C9—H9B	109.7	C74—C73—H73	119.9
H9A—C9—H9B	108.2	C72—C73—H73	119.9
N2—C6—C5	111.84 (11)	C55—C56—C57	120.82 (18)
N2—C6—H6A	109.2	C55—C56—H56	119.6
C5—C6—H6A	109.2	C57—C56—H56	119.6
N2—C6—H6B	109.2	C15—C24—C23	117.9 (2)
C5—C6—H6B	109.2	C15—C24—H24	121.1
H6A—C6—H6B	107.9	C23—C24—H24	121.1
C24—C15—C16	119.82 (17)	C54—C55—C56	119.44 (17)
C24—C15—C14	132.36 (17)	C54—C55—H55	120.3
C16—C15—C14	107.74 (12)	C56—C55—H55	120.3
			
C8—N1—C13—C17	−161.77 (10)	C52—C51—C5—C4	179.34 (13)
C12—N1—C13—C17	68.27 (14)	C52—C51—C5—C6	−3.5 (2)
C8—N1—C13—C14	85.87 (12)	O1—C4—C5—C51	−22.96 (19)
C12—N1—C13—C14	−44.09 (15)	C3—C4—C5—C51	155.19 (12)
C8—N1—C13—C3	−34.89 (12)	O1—C4—C5—C6	159.74 (13)
C12—N1—C13—C3	−164.86 (11)	C3—C4—C5—C6	−22.11 (17)
C8—C7—C71—C72	35.14 (18)	N1—C8—C9—C10	55.74 (16)
C3—C7—C71—C72	−87.39 (16)	C7—C8—C9—C10	169.61 (12)
C8—C7—C71—C76	−143.38 (13)	C2—N2—C6—C5	−54.29 (15)
C3—C7—C71—C76	94.09 (15)	C1—N2—C6—C5	−178.11 (13)
C1—N2—C2—C3	−163.22 (13)	C51—C5—C6—N2	−147.88 (13)
C6—N2—C2—C3	73.82 (13)	C4—C5—C6—N2	29.22 (18)
N1—C13—C17—C18	55.50 (18)	O2—C14—C15—C24	−5.5 (3)
C14—C13—C17—C18	174.76 (14)	C13—C14—C15—C24	−177.54 (19)
C3—C13—C17—C18	−61.52 (19)	O2—C14—C15—C16	171.13 (15)
N1—C13—C17—C16	−118.20 (12)	C13—C14—C15—C16	−0.94 (16)
C14—C13—C17—C16	1.06 (14)	C24—C15—C16—C21	1.7 (2)
C3—C13—C17—C16	124.78 (12)	C14—C15—C16—C21	−175.37 (14)
N2—C2—C3—C4	−61.97 (13)	C24—C15—C16—C17	178.83 (16)
N2—C2—C3—C7	174.16 (10)	C14—C15—C16—C17	1.73 (18)
N2—C2—C3—C13	56.87 (13)	C20—C21—C16—C15	178.90 (15)
O1—C4—C3—C2	−145.26 (12)	C22—C21—C16—C15	−0.4 (2)
C5—C4—C3—C2	36.60 (14)	C20—C21—C16—C17	2.1 (2)
O1—C4—C3—C7	−20.26 (17)	C22—C21—C16—C17	−177.24 (15)
C5—C4—C3—C7	161.60 (10)	C18—C17—C16—C15	−176.47 (13)
O1—C4—C3—C13	93.86 (14)	C13—C17—C16—C15	−1.81 (17)
C5—C4—C3—C13	−84.29 (13)	C18—C17—C16—C21	0.6 (2)
C71—C7—C3—C2	23.51 (15)	C13—C17—C16—C21	175.28 (13)
C8—C7—C3—C2	−104.61 (12)	C8—N1—C12—C11	59.07 (16)
C71—C7—C3—C4	−97.83 (13)	C13—N1—C12—C11	−173.91 (12)
C8—C7—C3—C4	134.04 (10)	C76—C71—C72—C73	0.3 (2)
C71—C7—C3—C13	145.25 (11)	C7—C71—C72—C73	−178.25 (15)
C8—C7—C3—C13	17.12 (12)	N1—C12—C11—C10	−55.75 (18)
N1—C13—C3—C2	132.33 (10)	C12—C11—C10—C9	55.3 (2)
C17—C13—C3—C2	−107.27 (13)	C8—C9—C10—C11	−54.83 (19)
C14—C13—C3—C2	11.23 (14)	C72—C71—C76—C75	−0.1 (2)
N1—C13—C3—C4	−109.69 (10)	C7—C71—C76—C75	178.50 (14)
C17—C13—C3—C4	10.71 (15)	C16—C21—C20—C19	−2.1 (2)
C14—C13—C3—C4	129.22 (11)	C22—C21—C20—C19	177.08 (19)
N1—C13—C3—C7	9.40 (12)	C57—C52—C53—C54	2.6 (3)
C17—C13—C3—C7	129.80 (11)	C51—C52—C53—C54	178.97 (18)
C14—C13—C3—C7	−111.69 (11)	C53—C52—C57—C56	−2.1 (3)
C5—C51—C52—C53	141.99 (17)	C51—C52—C57—C56	−178.41 (18)
C5—C51—C52—C57	−41.8 (2)	C71—C76—C75—C74	0.1 (3)
C16—C17—C18—C19	−3.2 (2)	C21—C20—C19—C18	−0.4 (3)
C13—C17—C18—C19	−176.42 (14)	C17—C18—C19—C20	3.2 (2)
N1—C13—C14—O2	−55.56 (18)	C76—C75—C74—C73	−0.3 (3)
C17—C13—C14—O2	−172.32 (14)	C24—C23—C22—C21	1.3 (4)
C3—C13—C14—O2	59.57 (18)	C20—C21—C22—C23	179.7 (2)
N1—C13—C14—C15	116.69 (12)	C16—C21—C22—C23	−1.1 (3)
C17—C13—C14—C15	−0.07 (14)	C52—C53—C54—C55	−0.7 (4)
C3—C13—C14—C15	−128.18 (12)	C75—C74—C73—C72	0.5 (3)
C12—N1—C8—C9	−59.48 (15)	C71—C72—C73—C74	−0.5 (3)
C13—N1—C8—C9	169.06 (11)	C52—C57—C56—C55	−0.1 (3)
C12—N1—C8—C7	177.93 (11)	C16—C15—C24—C23	−1.5 (3)
C13—N1—C8—C7	46.47 (12)	C14—C15—C24—C23	174.78 (19)
C71—C7—C8—N1	−166.60 (10)	C22—C23—C24—C15	0.0 (4)
C3—C7—C8—N1	−37.62 (12)	C53—C54—C55—C56	−1.6 (4)
C71—C7—C8—C9	74.61 (15)	C57—C56—C55—C54	2.0 (4)
C3—C7—C8—C9	−156.41 (11)		

### Hydrogen-bond geometry (Å, º)

**Table:**

*D*—H···*A*	*D*—H	H···*A*	*D*···*A*	*D*—H···*A*
C2—H2*B*···O2	0.97	2.35	2.9329 (18)	118
